# Correction to “N-Heterocyclic Carbene-Carbodiimide (NHC-CDI) Betaines as Organocatalysts for β-Butyrolactone Polymerization: Synthesis of Green PHB Plasticizers with Tailored Molecular Weights”

**DOI:** 10.1021/acscatal.4c01882

**Published:** 2024-04-09

**Authors:** David Sánchez-Roa, Valentina Sessini, Marta E. G. Mosquera, Juan Cámpora

**Affiliations:** † Departamento de Química Orgánica y Química Inorgánica, Instituto de Investigación en Química “Andrés M. del Río” (IQAR) Universidad de Alcalá, Campus Universitario, Alcalá de Henares, Madrid 28871, Spain; ‡ Instituto de Investigaciones Químicas, CSIC-Universidad de Sevilla, Sevilla 41092, Spain

**Corrections to the Acknowledgments**

In the original paper, the Acknowledgments on page 2499 were incorrect as published. That section should have read as follows:

**Acknowledgments**: This work was supported by the Grants PID2021-122708OB-C31 and PID2021-128392NB-100 funded by the MICIU/AEI/10.13039/501100011033 and ERDF/EU; the Grant TED2021-130871B–C22 funded by MICIU/AEI/10.13039/501100011033 and EU/PRTR; and the Thematic Network “OASIS” RED2022-134074-T funded by MICIU/AEI/10.13039/501100011033. The authors also thank the Comunidad de Madrid (No. EPU-INV/2020/001), the Junta de Andalucía (No. P20_00104), and the University of Alcalá (No. UAH-AE-2017-2). V.S. would like to thank the Ministerio de Ciencia e Innovación (Spain) and the European Community (Grant No. RYC2021-033921-I) for the financial support. D.S.R. thanks the Spanish Government for a Predoctoral Fellowship. We gratefully acknowledge Dra. Lucía Álvarez Miguel (UAH) for her assistance in the X-ray data collection, and Dra. Gloria Gutiérrez Alcalá (Mass Spectrometry Service, IIQ) for her assistance in sample preparation and recording ESI-MS spectra.

